# In Vitro Safety Assessment of In-House Synthesized Titanium Dioxide Nanoparticles: Impact of Washing and Temperature Conditions

**DOI:** 10.3390/ijms24129966

**Published:** 2023-06-09

**Authors:** Aliyah Almomen, Nasser B. Alsaleh, Ahmed Mohamed El-Toni, Mohamed A. EL-Mahrouky, Adel Ali Alhowyan, Musaed Alkholief, Aws Alshamsan, Nitish Khurana, Hamidreza Ghandehari

**Affiliations:** 1 Department of Pharmaceutical Chemistry, College of Pharmacy, King Saud University, Riyadh 11491, Saudi Arabia; 2 Department of Pharmacology and Toxicology, College of Pharmacy, King Saud University, Riyadh 11451, Saudi Arabia; nbalsaleh@ksu.edu.sa; 3 King Abdullah Institute for Nanotechnology, King Saud University, Riyadh 11451, Saudi Arabia; aamohammad@ksu.edu.sa; 4 Nanomaterials and Nanotechnology Department, Central Metallurgical Research and Development Institute (CMRDI), Cairo 11421, Egypt; 5 Soil Science Department, College of Food and Agriculture Science, King Saud University, Riyadh 11451, Saudi Arabia; 6 Department of Pharmaceutics, College of Pharmacy, King Saud University, Riyadh 11451, Saudi Arabia; adel-ali@ksu.edu.sa (A.A.A.); malkholief@ksu.edu.sa (M.A.);; 7 Department of Molecular Pharmaceutics, University of Utah, Salt Lake City, UT 84112, USAhamid.ghandehari@pharm.utah.edu (H.G.); 8 Utah Center for Nanomedicine, University of Utah, Salt Lake City, UT 84112, USA; 9 Department of Biomedical Engineering, University of Utah, Salt Lake City, UT 84112, USA

**Keywords:** engineered nanomaterials, TiO_2_ toxicity, nanotoxicology, RAW 264.7, HEK-293

## Abstract

Titanium dioxide nanoparticles (TiO_2_ NPs) have been widely used in food, cosmetics, and biomedical research. However, human safety following exposure to TiO_2_ NPs remains to be fully understood. The aim of this study was to evaluate the in vitro safety and toxicity of TiO_2_ NPs synthesized via the Stöber method under different washing and temperature conditions. TiO_2_ NPs were characterized by their size, shape, surface charge, surface area, crystalline pattern, and band gap. Biological studies were conducted on phagocytic (RAW 264.7) and non-phagocytic (HEK-239) cells. Results showed that washing amorphous as-prepared TiO_2_ NPs (T1) with ethanol while applying heat at 550 °C (T2) resulted in a reduction in the surface area and charge compared to washing with water (T3) or a higher temperature (800 °C) (T4) and influenced the formation of crystalline structures with the anatase phase in T2 and T3 and rutile/anatase mixture in T4. Biological and toxicological responses varied among TiO_2_ NPs. T1 was associated with significant cellular internalization and toxicity in both cell types compared to other TiO_2_ NPs. Furthermore, the formation of the crystalline structure induced toxicity independent of other physicochemical properties. Compared with anatase, the rutile phase (T4) reduced cellular internalization and toxicity. However, comparable levels of reactive oxygen species were generated following exposure to the different types of TiO_2,_ indicating that toxicity is partially driven via non-oxidative pathways. TiO_2_ NPs were able to trigger an inflammatory response, with varying trends among the two tested cell types. Together, the findings emphasize the importance of standardizing engineered nanomaterial synthesis conditions and evaluating the associated biological and toxicological consequences arising from changes in synthesis conditions.

## 1. Introduction

Intensive research efforts have been undertaken for the development of novel engineered nanomaterials (ENMs) and advanced materials, with numerous applications across different industry sectors [1]. Due to their unique physicochemical properties, titanium dioxide nanoparticles (TiO_2_ NPs) have been perceived as an attractive type of ENM for various applications including cosmetics, water purification, food additives, and nanomedicine [2]. TiO_2_ NPs are one of the most abundantly produced ENMs and they are commonly used in consumer products [3]. Such vast use of TiO_2_ NPs, particularly with a wide range of physicochemical properties, raises human safety concerns [4,5,6]. Indeed, recent regulations by France and the European Commission have been imposed for the use of TiO_2_ NPs (E171) in food [6]. This underscores the critical outcomes of nanotoxicological research over the past few years and points to the important role of the careful and thorough assessment of ENM biological and toxicological responses, including those nanoparticles that are considered generally inert and safe such as TiO_2_ NPs.

Exposure to a variety of metal and metal oxide ENMs, including TiO_2_ NPs, has been shown to be associated with toxicological manifestations in healthy individuals as well as the exacerbation of existing pathological conditions including cardiovascular, cancer, neurodegenerative, and immunological disorders [7,8,9,10,11]. For instance, inhalation exposure to TiO_2_ NPs has been shown to be associated with lung deposition, inflammation, cardiovascular injury, and microcirculation dysfunction [12,13]. Accumulated efforts by the nanotoxicological community over the past two decades have helped with the understanding and correlation of the physicochemical properties of TiO_2_ NPs (e.g., size, shape, surface charge, functionalization, etc.) in regard to their biological and toxicological responses. Nevertheless, predicting the biological and toxicological consequences of TiO_2_ NPs, particularly within a safety-by-design context, remains a major challenge [14]. To date, there remains uncertainty and discrepancy with regard to the physicochemical properties that are essentially responsible for driving the toxicity of TiO_2_ NPs [15,16,17,18,19,20,21,22,23]. For instance, it is yet unclear which crystalline form (i.e., rutile vs. anatase) of TiO_2_ NPs is associated with more toxicity [17,18,23,24,25]. This is because nano–bio interactions are complex and multifactorial and depend on the net outcome of interrelated factors, including physicochemical properties (chemical identity), bio-corona formation (biological identity), and the nature of the exposed tissue and cell type [14].

Titanium oxide has been synthesized by various approaches such as hydrothermal, solvothermal, micro-emulsion, sono-chemical, microwave-assisted, and sol-gel approaches [26,27,28,29,30,31]. The sol-gel approach is considered one of the simplest methods for the synthesis of TiO_2_ NPs. In this approach, titanium alkoxide is hydrolyzed and condensed to produce a network of titanium oxide. However, control over the shape (i.e., spherical morphology) and size of TiO_2_ NPs is quite difficult because of the fast hydrolysis of the titanium alkoxide precursor as well as their sensitivity to moisture and heat [32]. A possible way to retard the hydrolysis and condensation rates of titanium alkoxide is through the chemical modification of metal alkoxides with alcohols, acids or bases, and chelating agents [32]. A previous report employed a kinetics-controlled approach for the Stöber method to construct a porous TiO_2_ shell around different types of cores [33,34]. However, the kinetics-controlled Stöber method was not utilized for the formation of TiO_2_ NPs. Doping TiO_2_ with metal and non-metal has been considered as an effective way of tuning its band gap. On the other hand, the intrinsic defect formation of TiO_2_ for band gap engineering has been accomplished by electrochemical reduction and high-energy particle bombardment, such as with a photon beam and H2 plasma or electron beam [31]. Nevertheless, the utilization of washing conditions as a tool for the band gap and optical properties of the produced TiO_2_ NPs has not been reported before.

In this work, we evaluated the in vitro safety of in-house synthesized TiO_2_ NPs with variations in the synthesis conditions in phagocytic (RAW 264.7) and non-phagocytic (HEK-239) cell models. The aim of the present study was to assess the impact of changing the washing and temperature conditions during the synthesis of TiO_2_ NPs via the Stöber method on in vitro toxicity. Because of the critical role of TiO_2_ NP physicochemical properties in dictating nano–bio interactions, we extensively characterized the synthesized TiO_2_ NPs, including their size, surface area, surface charge, shape, crystalline phase composition, and band gap. Our results indicate that variation in TiO_2_ NP washing and temperature treatment resulted in cell type-dependent differences in biological and toxicological consequences following exposure to the TiO_2_ NPs (T1–4).

## 2. Results

### 2.1. Synthesis and Characterization of TiO_2_ NPs

**Figure 1:**
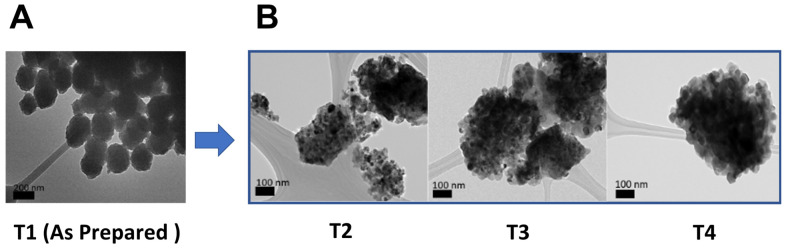
TEM images of TiO_2_ samples (**A**) as-prepared T1 and (**B**) T1 in higher magnifications and after different washing and calcination conditions (T2, T3, and T4).

TEM images were acquired to understand the impact of calcination temperature and washing conditions on the morphology of TiO_2_ NPs (Figure 1A,B). T1 demonstrated the T1 NPs that were prepared using the Stöber method. Here, titanium alkoxide was hydrolyzed under basic conditions in an ethanolic solution. The formed NPs retained an amorphous character, were round in shape, and had a size range of 220.5 ± 18.2 nm in diameter. When TiO_2_ was washed with ethanol and heat-treated at 550 °C (Figure 1B(T2)), the NPs lost their round morphology seen earlier with T1, and the resulting NPs were irregular in shape with a size range of 150.9 ± 20.4 nm. Moreover, T2 NPs agglomerated into small clusters, which could be attributed to grain growth during the heat-treatment process. The water washed sample calcined at 550 °C (T3) (Figure 1B(T3)) resulted in TiO_2_ NPs with an irregular shape, crystalline character, and a size range of 169.8 ± 26.3 nm. Elevating the heat-treatment temperature to 800 °C for the water washed sample (Figure 1B(T4)) did not affect the shape of the NPs but caused the size to increase to 232.4 ± 19.7 nm. It seems that the particle size was affected by the type of washing solution used. The ethanol used during heat treatment decomposed to carbon residues that acted as reductants for TiO_2_ NPs, slowing down the crystallization process, as evidenced by the small particle size of the T2 samples [35]. On the contrary, the T3 and T4 samples were water washed and there was no carbon residue present to affect the crystallization of TiO_2_ NPs. Therefore, the range of particle size in T1, T3, and T4 was larger than in particles washed with ethanol.

To shed light on the impact of the washing and calcination conditions on the phase change of TiO_2_ NPs samples, XRD measurements were conducted under different conditions and the spectra are shown in Figure S1. Sample T1 showed an amorphous character as seen from the broad peak at 2θ of 28. After washing T1 with ethanol and then heat-treating at 550 °C, it can be seen that a crystalline pattern was obtained which could be assigned to the anatase phase (T2). Upon washing the sample with water and performing heat treatment at 550 °C, the anatase phase was formed but with a high peak intensity referring to more growth of the crystallite size of the anatase phase, T3. Finally, increasing the heat treatment temperature to 800 °C for the water washed sample led to the formation of a mixture of anatase and rutile phase, T4. The anatase phase usually started to form at 400 °C and, therefore, a well-established phase can be seen at 550 °C. On the other hand, an anatase to rutile transformation may begin to take place at 700 °C and completely transform at 900 °C [36]. However, a noticeable difference in the peak intensity of the anatase phase of samples T2 and T3 indicated the impact of changing solvent on the phase growth. The low anatase peak intensity for the ethanol washed sample (i.e., T2) can be attributed to the fact that the ethanol in the sample started to decompose to form carbon residues that hindered the crystallization of TiO_2_ and anatase phase formation due to the enhancement of oxygen vacancies or slow crystallization processes [37]. However, by the end of the calcination process, the carbon no longer existed as a separate residue, instead, it doped TiO_2_ nanoparticles. The carbon dopant exists as either carbonate-like or graphite-like species which are usually located in the interstitial positions of the TiO_2_ lattice [38].

**Figure 2:**
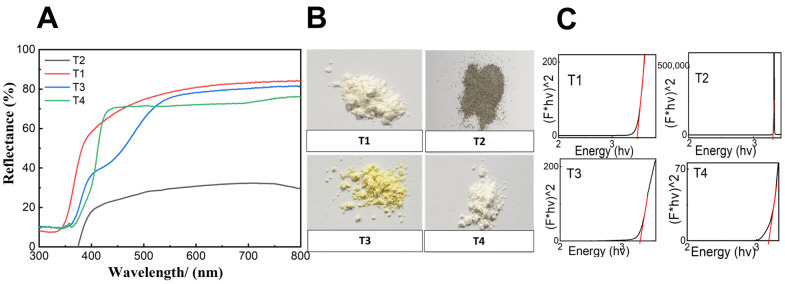
(**A**) Diffuse reflectance spectra, (**B**) colors, and the (**C**) Kubelka–Munk function band gap of the TiO_2_ samples formed from different washing and calcination conditions.

A diffuse reflectance measurement was conducted to investigate the change in the band gap of the titania samples by altering the washing and calcination conditions (Figure 2A). Furthermore, the change in the band gap will lead to a change in the color of the produced samples. It was clear that the reflectance spectra changed for the as-prepared sample and other ones prepared by ethanol and water (calcined at 550 and 800 °C). The T1, T3, and T4 samples showed similar reflectance ability in the visible light region from 400 to 800 nm but their UV-cut off varied from manipulating the washing and calcination conditions (Figure 2A). However, the T2 sample possessed much lower reflectance ability and UV-cut off ability. The T1 and T4 samples had a similar white color while T2 showed a gray color and T3 demonstrated a yellow color (Figure 2B). The band gap was calculated according to the Kubelka–Munk function (Figure 2C) and its values are mentioned in Table 1. The as-prepared sample showed the highest surface area, zeta-potential, and band gap. On the other hand, T4 showed the lowest surface area, zeta-potential, and band gap.

### 2.2. TiO_2_ NPs Affect the Cell Viability and Membrane Integrity of Phagocytic and Non-Phagocytic Cells

**Figure 3:**
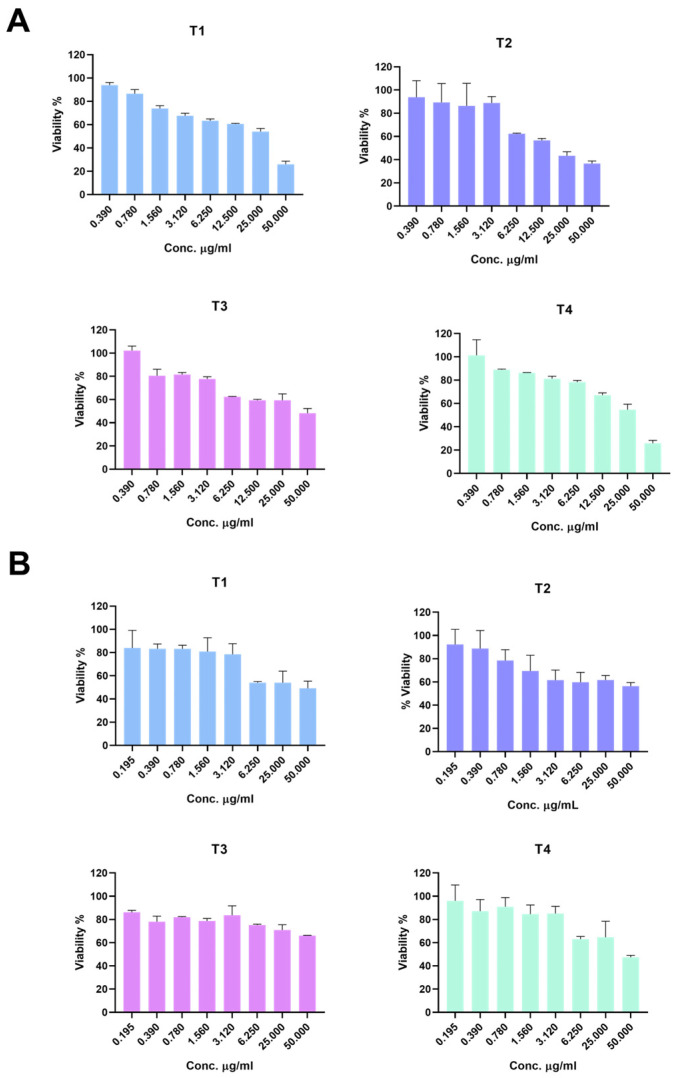
Cytotoxicity of T1–T4 TiO_2_ NPs in (**A**) RAW 264.7 and (**B**) HEK-293 cells after 24 h of exposure. Viabilities were above 50% in the lower concertation of TiO_2_ NPs and about 50% or less with the highest concertation used (50 µg/mL).

To assess the in vitro safety of the prepared TiO_2_ NPs, the cell viability of RAW 264.7 and HEK-293 cells was evaluated after exposing the cells to prob sonicated samples of T1–4 at a range of concentrations (50–0.195 µg/mL) [39]. The MTT results showed that the viability of cells was above 50% with most T1–4 concentrations (Figure 3A,B and Figure S2A,B), and no significant difference between treatment groups was found when cells were treated at a 0.78 µg/mL nanoparticle concentration. The viability of RAW 264.7 cells at 6.25 µg/mL depicted a significant difference between cells treated with T4 in comparison to T1–3. The cell viability was significantly different between all treatment groups when the cells were treated with a 50 µg/mL nanoparticle concentration, with the exception of cells treated with T1 and T4 (the difference was insignificant). In the HEK-293 cells, T3 showed the highest viability in doses of 6.25 or 50 µg/mL, which was statistically higher than the other TiO_2_ samples, and no significant difference was found in viability between T1, T2, and T4.

**Figure 4:**
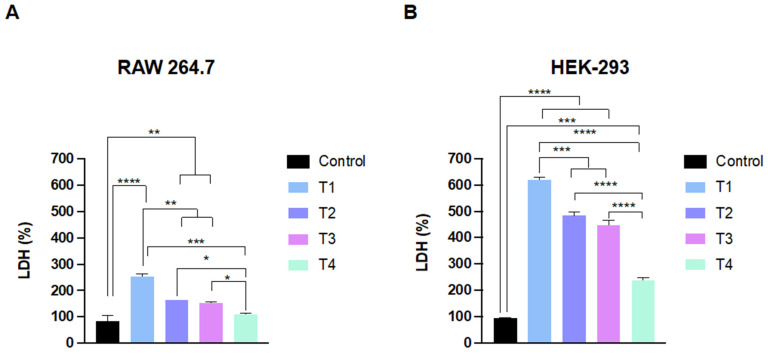
LDH immunoassay in RAW 264.7 (**A**) and HEK-293 cells (**B**). The cells exhibited a significant increase in LDH release after exposure to TiO_2_ NPs. Data are represented as the mean ± SD (*n* = 3). Statistical significance was obtained with *p*-values ≤ 0.05, where * *p* ≤ 0.05, ** *p* ≤ 0.01, *** *p* ≤ 0.001, and **** *p* < 0.0001.

The LDH assay results showed that the increase in LDH release in RAW 264.7 was about 2.5 times with T1, at least 1.5 times with T2 and T3, and there was no significant difference between the control and T4 treated group (Figure 4A). In the HEK-293 cells, there was a significant difference in LDH release between the control and TiO_2_ NP-treated cells and was pronounced with T1, which was significantly higher than other TiO_2_ NP-treated groups (Figure 4B).

### 2.3. Cellular Internalization of the Nanoparticles

**Figure 5:**
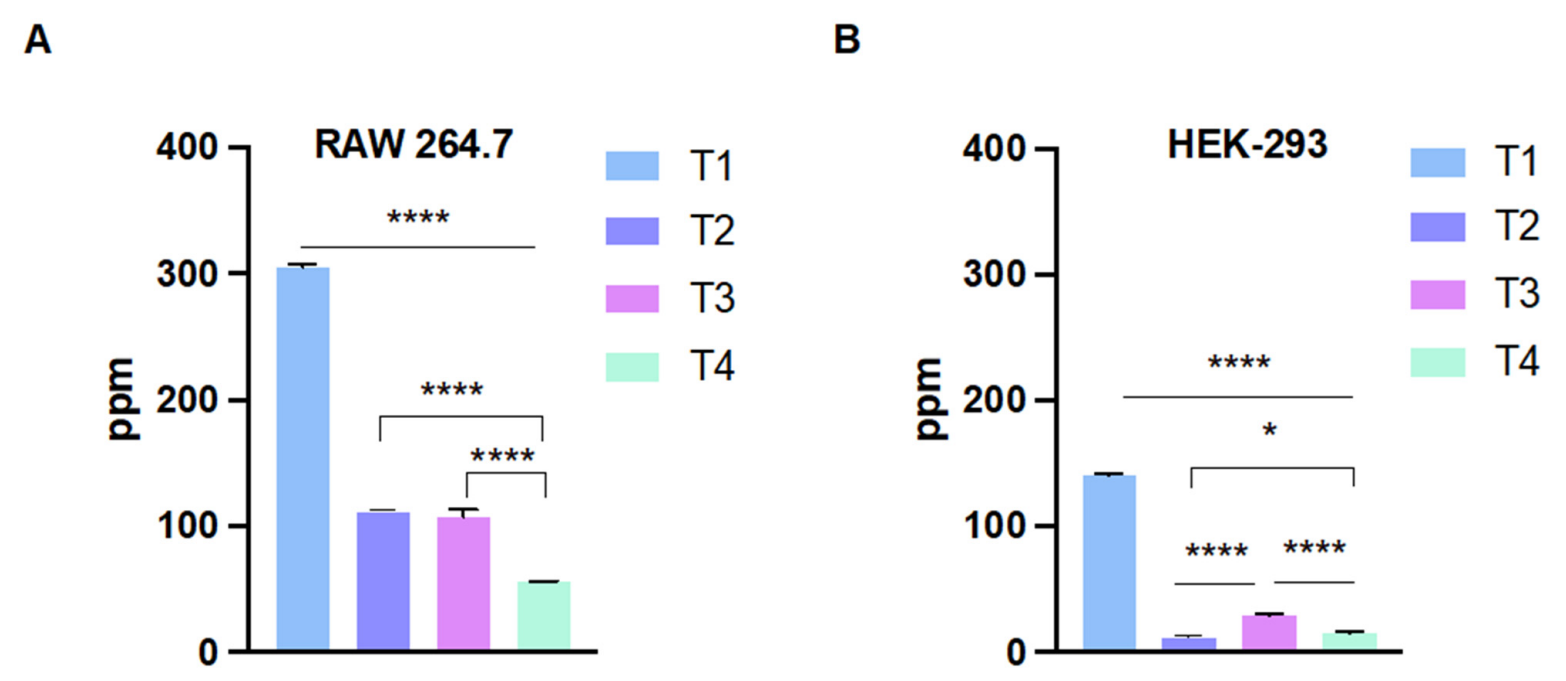
Cellular uptake of NPs in RAW 264.7 (**A**) and HEK-293 (**B**) cells as analyzed by ICP-MS using ^51^Sb as an internal standard. T1 shows the maximum increase in cellular uptake in both cell lines compared to other TiO_2_ NPs. Data are represented as the mean ± SD (*n* = 3). Statistical significance was obtained with *p*-values ≤ 0.05, where * *p* ≤ 0.05, and **** *p* < 0.0001.

TiO_2_ NP cellular internalization and association were quantified in RAW 264.7 and HEK-239 cells using ICP-MS following exposure to TiO_2_ NPs at 6.25 µg/mL for 4 h. The results demonstrated that the TiO_2_ NPs were internalized to different extents (Figure 5). The cellular internalization was significantly higher for the T1 TiO_2_ NPs in both cell types in comparison with the other TiO_2_ NPs. T1 and T2 TiO_2_ NPs had similar internalization levels in the RAW 264.7 cells (Figure 5A), however, the internalization of T2 was significantly less compared to T3 in the HEK-239 cells (Figure 5B). Finally, T4 TiO_2_ NPs had the least cellular internalization level in both cell types. The data also showed that the RAW 264.7 cells had more levels of internalized TiO_2_ NPs compared to the HEK-239 cells, which is expected given their primary phagocytic function. Images from confocal microscopy confirmed the uptake of TiO_2_ NPs and showed the colocalization of FIT-C labeled nanoparticles (green) and lysotracker (red) in both RAW 294.7 and HEK-293 cells, indicating that endocytosis might possibly be the main route of TiO_2_ internalization (Figure S3A,B).

### 2.4. Generation of Intracellular Reactive Oxygen Species (ROS)

**Figure 6:**
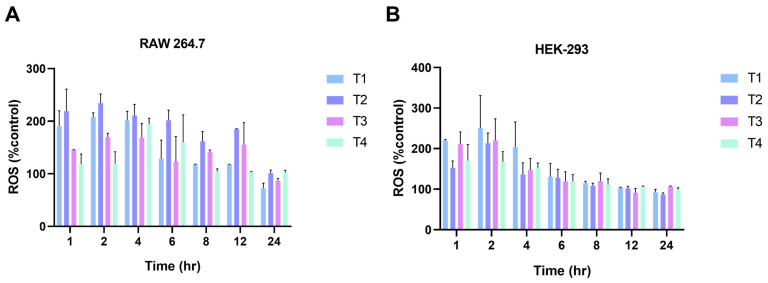
TiO_2_ NPs induce ROS generation in RAW 269.7 (**A**) and HEK-293 (**B**) cells over time. Data indicate a % increase in ROS production relative to the control. Maximum ROS production was found with T1 and T2 within 2 h, and in 3 h with T3 and T4 in RAW 264.7 cells. In HEK-293 cells, ROS maximum production was found within 2 h of T1–T4 NP exposure with no significant difference between treatment groups.

To evaluate the oxidative potential of the TiO_2_ NPs, the generation of ROS was measured after exposing the cells to 6.25 μg/mL of TiO_2_ NPs over time (1, 2, 4, 6, 8, 12, and 24 h). Data indicated that all TiO_2_ NPs resulted in ROS generation in both cell types, with the maximum increase occurring at around 2 and 4 h and with T1 and T2 TiO_2_ NPs. Specifically, significant differences were found between T1 and T3 as well as between T2 and both T3 and T4, and at 4 h with T3 and T4 post exposure (Figure 6A). The HEK-293 cells exhibited a maximum increase as well in ROS within 2 h of exposure to TiO_2_ NPs, with no significant difference between treatment groups (Figure 6B). Table S1 depicts the significant differences between treatment groups.

### 2.5. Release of Inflammatory Mediators

**Figure 7:**
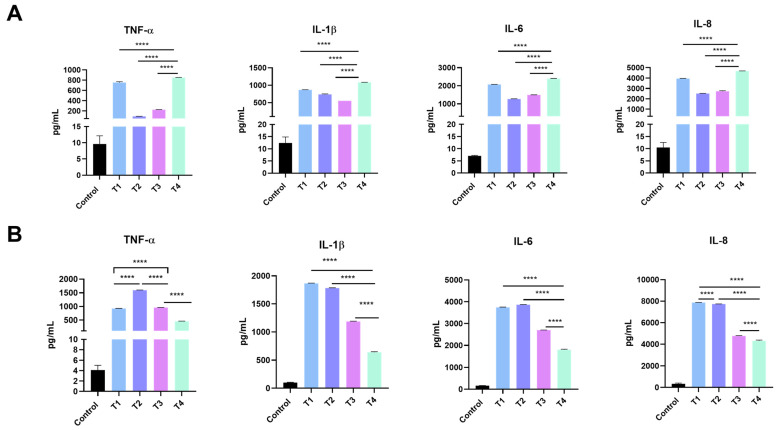
Inflammatory cytokines levels in (**A**) RAW 264.7 and (**B**) HEK-293 cells after exposure to TiO_2_ NPs in comparison to the control. T1 and T4 show the highest potential to increase IL-1β, IL-6, IL8, and TNF-α levels in RAW 264.7. T1 and T2 induce the highest increase in IL-1β, IL-6, IL8, and TNF-α in HEK-293 cells. Data are represented as the mean ± SD (*n* = 3). Statistical significance was obtained with *p*-values ≤ 0.05, where **** *p* < 0.0001.

A panel of inflammatory cytokines was evaluated in RAW 264.7 and HEK-293 cells after being exposed to 6.25 g/mL of TiO_2_ NPs for 24 h. In RAW 264.7 cells, T1 and T4 showed the highest increase in levels of tumor necrosis factor-alpha (TNF-α), IL-1β, IL-6, and IL8, IL-1α, interferon-gamma (IFN-γ), granulocyte-macrophage colony-stimulating factor (GM-CSF), and monocyte chemotactic and activating factor (*MCAF*) (Figure 7A and Figure S4A), while in the HEK-293 cells, T1 and T2 induced the highest increase in cytokine levels (Figure 7B and Figure S4B).

## 3. Discussion

The use and incorporation of inorganic engineered nanomaterials (ENMs) into biomedical and consumer products are vastly expanding [1]. This is mainly driven by the biomedical potential and high tunability of the physicochemical properties of inorganic ENMs [1]. However, one caveat is the potential adverse responses of inorganic ENMs at the nano level compared to the bulk level [40]. Indeed, the nanotoxicology community has been striving to understand the bio-physicochemical interaction of ENMs and the driving factors of toxicity when including the materials’ inherent physicochemical properties [41]. These efforts have established the basics and it is now possible to anticipate the biological and toxicological outcomes of inorganic ENMs based on their physicochemical properties such as size, shape, charge, and surface functionalization [6,41]. However, there remains uncertainty and inconsistency between studies with regard to the correlation between material physicochemical properties and their biological and toxicological effects, including TiO_2_ NPs [42,43].

TiO_2_ NPs are among the most widely used ENMs across multiple industries, including food and cosmetics [2]. Furthermore, extensive efforts are being put forth toward the development of novel applications, including in nanomedicine, and continuous efforts are being undertaken to better improve the properties of TiO_2_ NPs [44]. Indeed, recent studies have demonstrated that TiO_2_ NPs, in their drug-free form, are promising in the management of cancer as they are capable of generating ROS within the tumor microenvironment [45,46]. Thus, human exposure to TiO_2_ NPs will undeniably increase in the future, and hence, the safety assessment of TiO_2_ NPs is critically warranted [6,47]. There remains a need to better understand the biological and toxicological responses following exposure to TiO_2_ NPs, which is not only key for addressing their safety concerns but also important for exploiting TiO_2_ NPs in nanomedicine and biomedical applications [5,10,43,44]. Therefore, this study aimed to assess the toxicity and delineate the correlation between the physicochemical properties and biological response of TiO_2_ NPs synthesized under different washing conditions.

The protocols used in the synthesis of ENM typically vary, whether in industry or academic laboratories. This leads to variation in the physicochemical properties of ENMs and consequently may result in a drastic change in their biological and toxicological behavior. Therefore, it is of critical importance to understand and correlate synthesis conditions and resultant physicochemical properties with the corresponding biological and toxicological properties. In this study, TiO_2_ NPs (T1–4) were first synthesized by a kinetics-controlled Stöber method (T1; as-prepared sample) and then three different yields of TiO_2_ NPs (T2–4) were obtained by changing the washing condition (water or ethanol) and calcination temperature (at 550 °C or 800 °C). The TiO_2_ NPs were then extensively characterized to better correlate their physicochemical properties with biological behavior. Washing the as-prepared sample (T1) with ethanol along with heat treatment at 550 °C (T2) resulted in a dramatic change in the particle physicochemical properties from a round amorphous to an irregular crystalline structure with a large reduction in the particle surface area, charge, and band gap. In addition, the TiO_2_ NPs demonstrated the formation of small clusters attributed to grain growth during heat treatment. Similarly, washing with water while applying heat at 550 °C and 800 °C (T3 and T4) resulted in the formation of an irregular crystalline pattern with larger crystallite size and a reduction in the surface area and charge as well as band gap, albeit to a lesser extent compared with ethanol. It is also worth noting that increasing the heat temperature resulted in an increased particle size (T3 and T4) (Figure 1B). Similar findings have been found in recent reports where the calcination of TiO_2_ NPs resulted in a phase transition, increased crystallinity, and reduced band gap energy [25,48].

The viability study demonstrated that exposure to TiO_2_ NPs was associated with a dose-dependent reduction in cell viability in both RAW 264.7 and HEK-293 cells, with observed differences among the T1–4 TiO_2_ NPs (Figure 3). Although the size of the TiO_2_ NPs remained within a size range of about 150–250 nm in diameter, exposure to the T1 TiO_2_ NPs was associated with the most toxicity. This could be attributed to the increased surface area resulting in significant internalization into both cell types as demonstrated by the ICP-MS data (Figure 5). Indeed, a correlation between TiO_2_ NP’s surface area and toxicity has been reported before [17]. Upon washing and heat treatment, the cellular internalization of the TiO_2_ NPs (T2–4) was significantly lower compared to the T1 TiO_2_ NPs, as was the corresponding toxicity. Although shape has been reported previously to influence TiO_2_ NP toxicity, we do not believe it played a major role in the observed toxicity since all the TiO_2_ NPs were round-like in shape [49,50]. One key feature of the TiO_2_ NPs that played a role in their toxicity was their crystalline structure. The two major crystalline structures of TiO_2_ NPs are anatase and rutile, both of which are used in a variety of applications including cosmetics, paints, and food [2]. Although previous reports are inconsistent as to whether anatase or rutile is associated with more toxicity, they all demonstrated that a change in the crystalline structure influences the toxicity of TiO_2_ NPs [17,18,23,24,25]. In the present study, and despite such a large difference in the cellular internalization between the T1 and the other TiO_2_ NPs (T2–4), the difference in toxicity was found to be less in magnitude. Based on the previous literature, this may suggest that the crystallinity, crystalline structure formation, of the T2–4 TiO_2_ NPs was partially responsible for their toxicity [16,17,18]. Moreover, the cellular internalization data suggests that the toxicity of T2–4 TiO_2_ NPs might be due to the induction of cell membrane damage rather than cellular internalization. It is also worth mentioning that although previous studies showed conflicting data with regard to the relationship between the type of crystalline phase and toxicity, more evidence supports that the anatase phase is more associated with toxicity due to its ability to generate ROS [16,17,18,24,38,51]. We speculate from our data and previous reports that heat treatment at 800 °C and the resultant formation of the rutile crystalline phase in the T4 TiO_2_ NPs reduced cellular internalization and toxicity [15,20,24]. However, further work is needed to clarify the association between the crystalline form and the toxicity of TiO_2_.

The major paradigm of ENM toxicity is mediated via the formation of ROS either upon interaction with the cell membrane or following cellular internalization and the release of metal ions inside the lysosomes, which eventually leads to oxidative damage and cell death [40,41]. Therefore, we measured ROS formation over time as well as changes in lysosomal pH. The data revealed that exposure to all TiO_2_ NPs was associated with ROS generation at early time points, which then subsided over time (Figure 6). Despite the differences in the physicochemical properties of the TiO_2_ NPs and associated cellular internalization and toxicity, the ROS levels were relatively comparable between the T1–4 TiO_2_ NPs. These findings suggest that crystalline TiO_2_ NPs (T2–4), although not internalized to the same degree compared to amorphous TiO_2_ NPs (T1), were still able to generate ROS to a similar extent. Previous reports also failed to find a correlation between TiO_2_ NP physicochemical properties and ROS generation [17,24]. Moreover, one observation from the ROS data was a trend of increased ROS levels in T1 and T2 in the RAW 264.7 cells during the first two hours of exposure. Such a response may be explained by the rate of internalization of the TiO_2_ NPs. Another observation was that the ROS levels appeared to sustain for a longer time in the RAW 264.7 cells. One explanation could be the higher internalization and accumulation of the TiO_2_ NPs in RAW 264.7 cells, as evident in the ICP-MS data. It is also worth mentioning that the two cell models used to assess the toxicity in this study are phenotypically distinct, and hence, it was anticipated that the cells could behave differently following exposure to the TiO_2_ NPs and that there might be differences, both quantitative and qualitative, in their toxicological outcomes [52]. On the other hand, the results did not show a reduction in the fluorescence of the lysosomal probe, suggesting that lysosome-associated TiO_2_ NPs do not disrupt lysosomal function at a concentration of 6.25 µg/mL. Together, these findings suggest that TiO_2_ NP toxicity is in part independent of ROS generation and cellular accumulation. Interestingly, one study showed that rutile TiO_2_ NPs have a higher affinity to interact with proteins and phospholipids and, hence, might explain their higher toxicity, while anatase TiO_2_ NPs resulted in the increased disruption of mitochondrial function [24]. Such findings indicate that TiO_2_ NPs could induce toxicity through multiple mechanisms that are both ROS-dependent and independent.

Activation of the cellular inflammatory response is an important parameter of exposure and toxicity in response to ENM insult, and it represents phase two of the hierarchical oxidative stress model [40,53]. Ample evidence suggests that exposure to TiO_2_ NPs is associated with the activation of the cellular inflammatory response and reduction in cellular function [15,20,24,54,55,56,57,58,59]. The underlying mechanisms are not fully understood; however, accumulating research suggests that exposure to TiO_2_ NPs could activate pattern recognition receptors (e.g., toll-like receptors), downstream signaling pathways (e.g., NF-κB), and executor complexes (e.g., inflammasome), resulting in different organ-specific toxicities, for example, in the lung, liver, and brain [54,56,57,58]. Our study evaluated a panel of inflammatory markers to gain insight into the nature of inflammatory response following exposure to TiO_2_ NPs, including TNFα, IL-1α, IL-1β, IL-6, IL-8, IFNγ, GM-CSF, and MCAF (Figure 7 and Figure S4). The findings demonstrated that exposure to all TiO_2_ NPs resulted in activation of the cell inflammatory response in both cell models although with a different pattern. This difference in pattern goes in parallel with the difference in expression levels of LDH. Studies have shown that LDH levels have the potential to influence the levels of inflammatory cytokines in the body such as IL-6 and TNF-α [60]. LDH is an enzyme that participates in the production of energy in cells, and when its levels are elevated, it can be indicative of cell damage or death [61]. Inflammatory cytokines are then secreted as an immune response to infections and traumas [61]. For instance, higher LDH levels have been linked to more inflammatory cytokines and a more severe illness course in COVID-19 patients [62].

Exposure to T1 and T2 TiO_2_ NPs was associated with a significantly higher inflammatory response in the HEK-293 cells across all tested inflammatory mediators, whereas exposure to T1 and T4 TiO_2_ produced an increased inflammatory mediator release in the RAW 264.7 cells. The results of the T1 TiO_2_ NPs are consistent with other previous data (e.g., cell viability, uptake, etc.), however, in the case of T2 TiO_2_ NPs in the HEK-293 cells or T4 TiO_2_ NPs in the RAW 264.7 cells, the data are inconsistent. For instance, although T4 TiO_2_ NPs in the RAW 264.7 cells were associated with the least internalization and toxicity, they induced a stronger inflammatory response compared with the T2 and T3 TiO_2_ NPs. Such data demonstrates the complexity of biological and toxicological responses with regard to intercellular differences and inconsistencies with the other toxicological endpoints (e.g., viability and internalization). Additional studies are needed to investigate this phenomenon.

## 4. Materials and Methods

### 4.1. Nanoparticle Synthesis

Titanium (IV) butoxide, ammonium hydroxide, and ethanol were purchased from Sigma-Aldrich. All the chemicals were utilized without further purification. TiO_2_ NPs were prepared by the kinetics-controlled Stöber method [33,34]. In a glass bottle, 0.2 mL of ammonia solution (27%) was added to 50 mL of absolute ethanol, then the mixture was stirred (600 rpm) for 5 min. Thereafter, 0.2 mL of deionized water was added to the previous mixture. After homogenization for 5 min, 0.5 mL of Titanium (IV) butoxide was finally added and the solution was stirred (600 rpm) for 180 min at 65 °C. After the completion of the reaction, the washing step was conducted using absolute ethanol or water. The initially prepared sample was denoted as T1. The sample washed with ethanol and heat-treated at 550 °C was described as T2. Samples washed with water and heat treated at 550 and 800 °C were described as T3 and T4, respectively. The heat-treatment process was conducted in a capped porcelain crucible. The sample preparation conditions, band gap, surface, and zeta-potential and phase data are provided in Table 1.

### 4.2. Nanoparticle Characterization

X-ray diffraction pattern measurements were performed on the Bruker D8 Advance diffractometer with Cu-Kα radiation (35 kV, 30 mA) and a diffracted beam monochromator. The diffuse reflectance measurement was conducted using the integrating sphere of a Shimadzu UV-2550 UV/VIS spectrophotometer. The BET surface area measurement was conducted using Quantachrome nova 4200e. Prior to the measurement, the sample was degassed at 150 °C for 24 h. The zeta-potential measurement was conducted using a Malvern nanosizer. The TEM observation was conducted using a transmission electron microscope (TEM) operating at 200 kV (a JEOL JEM-2100F-UHR field-emission instrument) with a Gatan GIF 2001 energy filter and a 1k-CCD camera.

### 4.3. Cell Culture

RAW 264.7 and HEK-239 cells were obtained from the American Type Culture Collection (ATCC, Rockville, MD, USA). The cells were cultured according to ATCC instructions using standard aseptic techniques in Dulbecco’s Modified Eagle’s Medium (DMEM), supplemented with 10% fetal bovine serum (FBS) and 100 UI mL^−1^ penicillin G, 100 µg mL^−1^ streptomycin, and incubated at 37 °C in 5% carbon dioxide/95% air.

### 4.4. Cell Viability and Membrane Integrity

The cytotoxicity of TiO_2_ NPs was evaluated in RAW 264.7 (mouse peritoneal macrophages) and HEK-293 (embryonic human kidney epithelial) cells. The cells were seeded in a 96-well culture plate at a density of 1 × 10^4^ per well in 100 µL of culture media and incubated for 24 h. A serial dilution of TiO_2_ NPs at a concentration range of 0.195 to 50 µg/mL was prepared, probe sonicated for 3 min to disperse NP agglomerates, and then incubated with cells for 24 h [39]. A total of 20 µL of 2.5 mg/mL of 3-(4,5-dimethylthiazol-2-yl)-2,5-diphenyltetrazolium bromide (MTT) in PBS was added to the cells and they were further incubated for 4 h at 37 °C. The MTT solution was then completely removed and 100 µL of DMSO was added to solubilize the formazan crystals. Absorbance was measured at 540 nm using a Spectramax 250 microplate reader (Molecular device, San Jose, CA, USA). Cell viability (%) was calculated as the optical density (OD) of [treated cells/OD of non-treated cells] × 100. The concentration 6.25 μg/mL was used in subsequent experiments because it produced apparent but not overwhelming toxicity, with cellular viability ranging from ~50 to 80% following 24 h of exposure to the different TiO_2_ NPs (Figure S2).

To evaluate the cellular membrane integrity after TiO_2_ NPs treatment, the lactate dehydrogenase (LDH) assay was used (LDH cytotoxicity assay kit, Cyman Chemical, Ann Arbor, Michigan, USA) according to the manufacturer’s protocol. Briefly, RAW 264.7 and HEK-293 cells were seeded in a 6-well culture plate at a density of 1 × 10^5^ per well in 500 µL culture media and incubated for 24 h. The cells were then treated with 6.25 μg/ of TiO_2_ for 24 h. They were then centrifuged at 250× *g* for 7 min. The supernatant was withdrawn and 50 μL was diluted 1:1 with the reaction mixture and incubated for 30 min in the dark at room temperature. Finally, the absorbance was recorded at 490 nm with a reference wavelength of 610 nm using a UV spectrophotometer (Molecular device, San Jose, CA, USA). Spontaneous LDH release was detected from the untreated cells as a negative control.

### 4.5. Measurement of Intracellular Reactive Oxygen Species (ROS)

Intracellular ROS levels were measured spectrofluorometrically using the dichlorofluorescein (DCF) assay (ThermoFisher Scientific, Waltham, MA, USA). RAW 264.7 and HEK-293 cells were first cultured in 96-well plates (5 × 10^4^ cells/ well) for 24 h. The cells were then treated with 6.25 μg/mL of TiO_2_ NPs at different time points, subsequently washed with PBS twice, treated with 25 μM DCF-DA, and incubated in the dark for 30 min at 37 °C. The fluorescence intensity was measured at an excitation wavelength of 485 nm and emission wavelength of 528 nm using a fluorescence microplate reader (Molecular device, San Jose, CA, USA). Data were then presented as ROS% control (non-treated cells).

### 4.6. Cytokine Immunoassay

Cytokines were measured using a Multiplex Cytokine ELISA kit following the manufacturer’s protocol (MY BioSource, Multiplex Cytokine ELISA kit, San Diego, CA, USA). Briefly, cells were seeded at a density of 5 × 10^4^ cells per well in 96-well plates. The cells were then incubated with 6.25 μg/mL TiO_2_ for 24 h. Next, 50 μL of a biotin conjugate mixture was added to each well and then the plates were covered and incubated for 1 h at room temperature. The cells were then washed five times and blot dried. One hundred microliters of Avidin HRP conjugate mixture was then added to each well, mixed well, covered, and incubated for 1 h at room temperature. The plates were then washed, and 100 μL of substrate solution was added to each well, covered, and incubated for 15 min at room temperature. One hundred microliters of stop solution were then added and plates were measured within 10 min at 450 nm using a microtiter plate reader. The expression of the inflammatory cytokines was then compared to the results of the non-treated control cells.

### 4.7. Cellular Uptake Measured by Inductively Coupled Plasma Mass Spectrometry (ICP-MS)

RAW 264.7 and HEK-293 cells were treated with 6.25 μg/mL of TiO_2_ NPs for 4 h. The cells were gently washed with PBS and 250 μL of 0.25% trypsin was added to each well of HEK-293 cells or scraped, in the case of RAW 264.7 cells, to detach the cells. After detachment, the cells were centrifuged at 600× *g* and the cell pellets were washed with PBS thoroughly. After aspirating PBS, the pellets were dissolved in 70% nitric acid overnight and were then diluted to 2% before analyzing via ICP-MS. TiO_2_ NPs were quantified by ICP-MS using ^15^Sb as an internal standard with a limit of detection of 6 ppb.

### 4.8. Statistical Analysis

Statistical analyses were performed using GraphPad Prism 8.0.1 software (GraphPad Software Inc., San Diego, CA, USA). All experiments were conducted in triplicate unless indicated otherwise. Results are expressed as the mean ± SD, and *p* < 0.05 was considered as the threshold of significance. Analysis of variance (ANOVA) and Tukey’s multiple comparisons test were used when comparing three or more treatment groups.

## 5. Conclusions

The findings of this study indicated that the change in washing and temperature conditions in the synthesis of TiO_2_ NPs resulted in a cell type-specific variation in biological and toxicological responses. Most importantly, the consequent biological and toxicological responses of TiO_2_ NPs (e.g., reduced viability, ROS generation, inflammatory response, etc.) cannot be simply attributed to a single or couple of differences in the physicochemical properties of TiO_2_ NPs but rather to a combination of the TiO_2_ NP physicochemical properties. For instance, and as shown in this study, even when the particle size and zeta potential are relatively within range (i.e., T3 vs. T4 TiO_2_ NPs), the cellular internalization and toxicity varied significantly. Equally important is the complexity of biological responses. This has been demonstrated by the inflammatory mediator release data where the inconsistency between toxicological outcomes and inflammatory mediator release (i.e., in the RAW 264.7, the inflammatory mediator release of T4 TiO_2_ NPs was comparable to T1 TiO_2_ NPs although the internalization and toxicity varied significantly between the two TiO_2_ NPs). The data also showed a different pattern among the two cell models.

Furthermore, the findings also emphasize that even slight changes in the washing conditions of TiO_2_ NPs could largely influence cellular response and hence, the safety profile. It is worth mentioning that emerging research has also focused on studying the behavior of TiO_2_ NPs in different biological fluids to better understand the role and impact of bio-corona formation with regard to their physicochemical properties as another layer that could explain the TiO_2_ NP’s biological and toxicological responses [19,63,64,65]. Despite the potential of ENMs in nanomedicine and biomedical applications, including the use of TiO_2_ NPs in cancer therapy, it is crucial for the development of new TiO_2_ NPs for preclinical testing to extensively characterize their physicochemical properties and thoroughly understand their biological behavior and safety profile [45].

## Figures and Tables

**Table 1:** Characterization of the TiO_2_ NPs at different washing and temperature conditions.

Sample	Size * (nm)	Surface Area (m^2^/g)	Zeta-Potential (mV)	Band Gap (eV)	Crystalline Form
T1	220.5 ± 18.2	200.483	−44.9 ± 1.15	3.480	Amorphous
T2	150.9 ± 20.4	4.766	−7.39 ± 1.18	3.303	Anatase
T3	169.8 ± 26.3	10.458	−25.8 ± 0.70	3.299	Anatase (high intensity)
T4	232.4 ± 19.7	7.053	−27.1 ± 0.40	3.178	Anatase and rutile

* The size was determined using TEM by taking the average of 50 nanoparticles.

## Data Availability

Data are contained within the article or Supplementary Material.

## Supplementary Materials

The supporting information can be downloaded at: https://www.mdpi.com/article/10.3390/ijms24129966/s1.
