# Supplementary material for: In Vitro Safety Assessment of In-House Synthesized Titanium Dioxide Nanoparticles: Impact of Washing and Temperature Conditions
Source: Int J Mol Sci. 2023 Jun 9;24(12):9966. doi: 10.3390/ijms24129966 (PMC10298741; doi:10.3390/ijms24129966)
Supplement: Supplementary file 1 [file ijms-24-09966-s001.zip › ijms-2442342-supplementary.pdf]

In vitro Safety Assessment of in-house Synthesized Titanium Dioxide Nanoparticles: Impact of Washing and Temperature Conditions

Cellular colocalization

RAW 264.7 and HEK-293 cells were seeded in a density of 3×10^4 cells/well into Cellview 35-mm glass-bottom cell culture dishes with four compartments (Greiner Bio-One International GmbH) for 24 h. Cells were then treated with FITC-labeled TiO2 NPs (6.25 µg/mL), at 37°C for 1 4h. LysoTracker Red (50 nM; Molecular Probes; Thermo Fisher Scientific, Inc.) was added during the last 1 h at 37°C. Next, cells were visualized using an LSM 700 confocal laser scanning fluorescence microscope (Zeiss GmbH) equipped with a Plan-Apochromatic 63×/1.4 oil DIC (Zeiss GmbH). All images were acquired and processed using ZEN 2012. The object-based fluorescence intensity was measured using Image software.

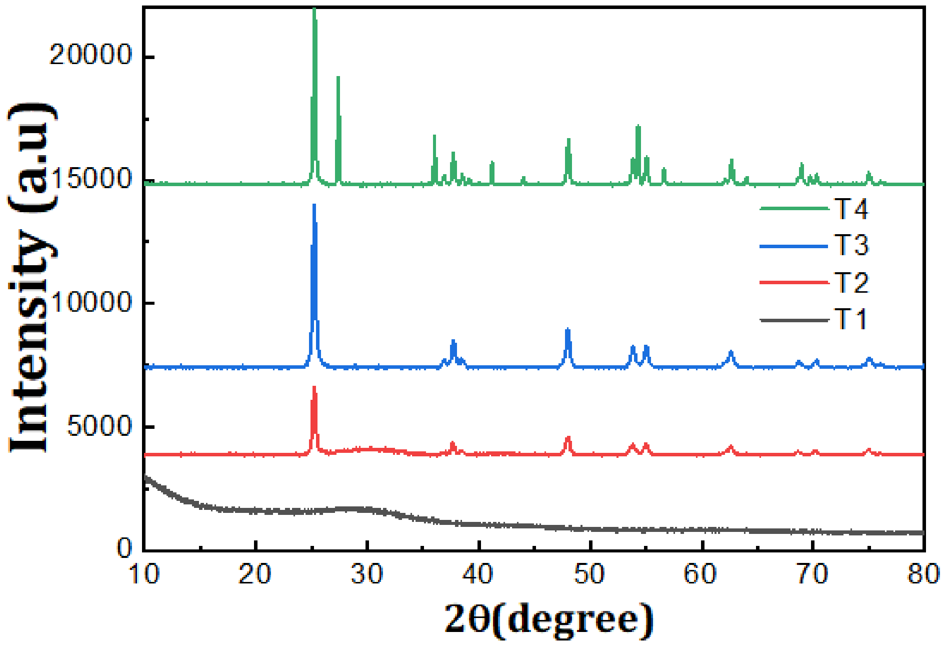

Figure S1. XRD spectra of TiO2 samples formed at different washing and calcination condition.

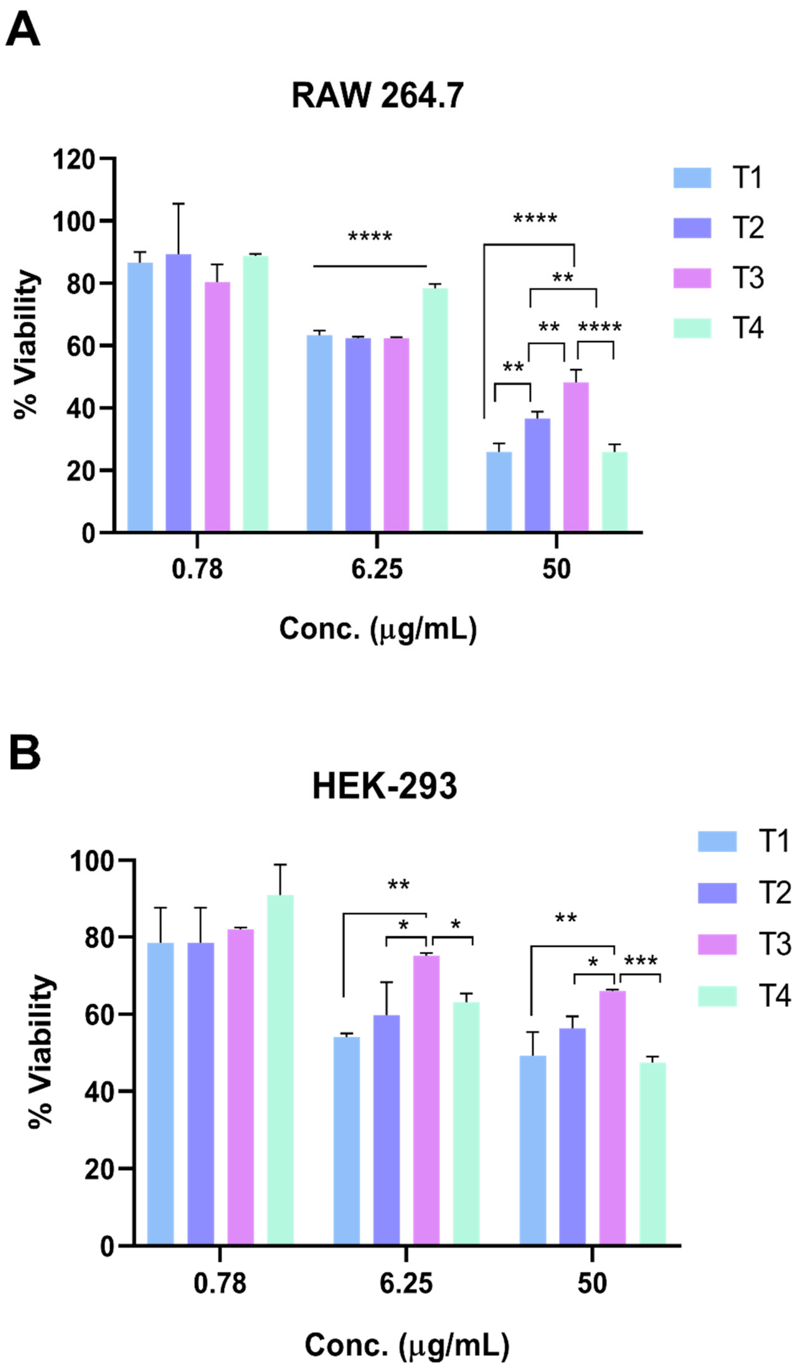

Figure S2. Cytotoxicity of T1-T4 TiO2 NPs in (A) RAW 264.7 and (B) HEK-293 cell in 24 hrs. Viabilities are above 50% low concertation of TiO2 NPs (0.78 and 6.26 µg/mL) and about 50% or less with the highest concertation used (50 µg/mL). Data are represented as mean ± SD (n = 3). Statistical significance was obtained with p-values ≤ 0.05, where * $p \leq 0.05$, ** $p \leq 0.01$, *** $p \leq 0.001$, and **** $p < 0.000$

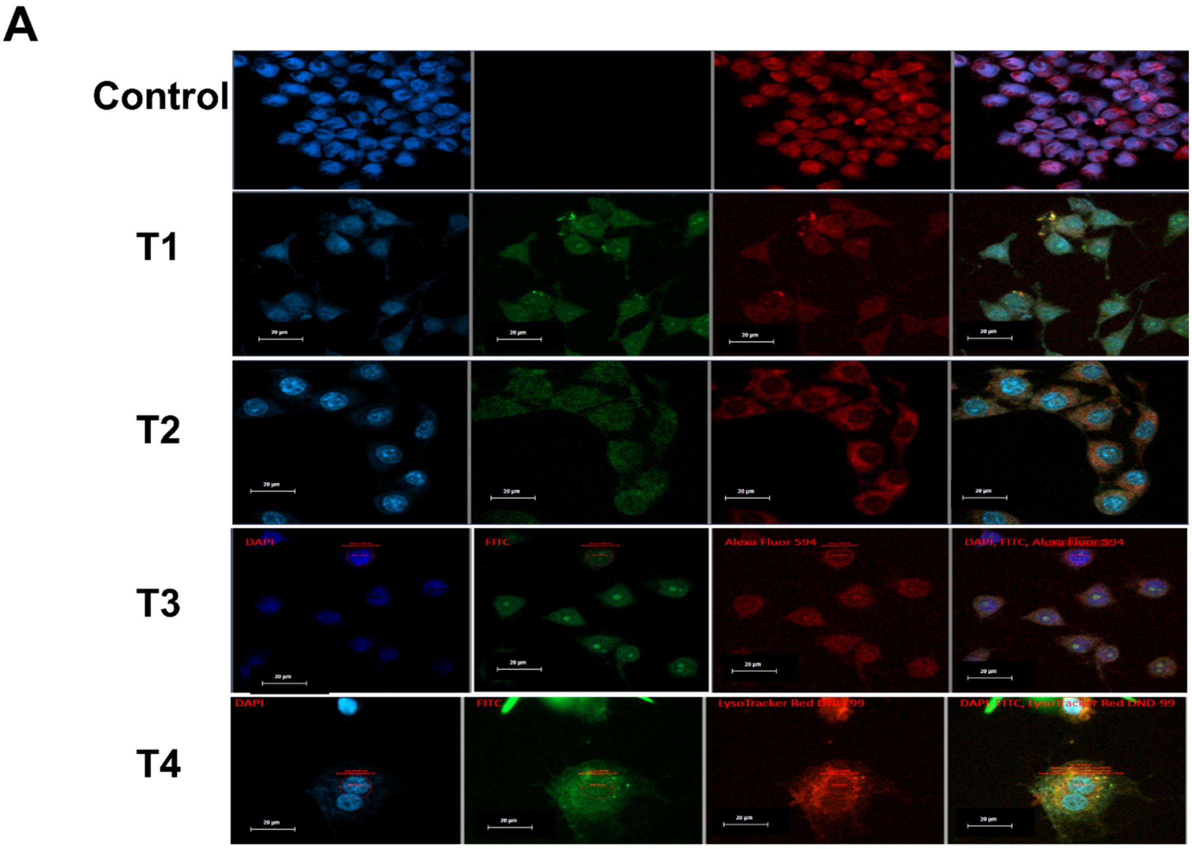
A
Control
T1
T2
T3
T4
DAPI
FITC
Alexa Fluor 594
DAPI, FITC, Alexa Fluor 594
LysoTracker Red DND-99
DAPI, FITC, LysoTracker Red DND-99
20 μm

B

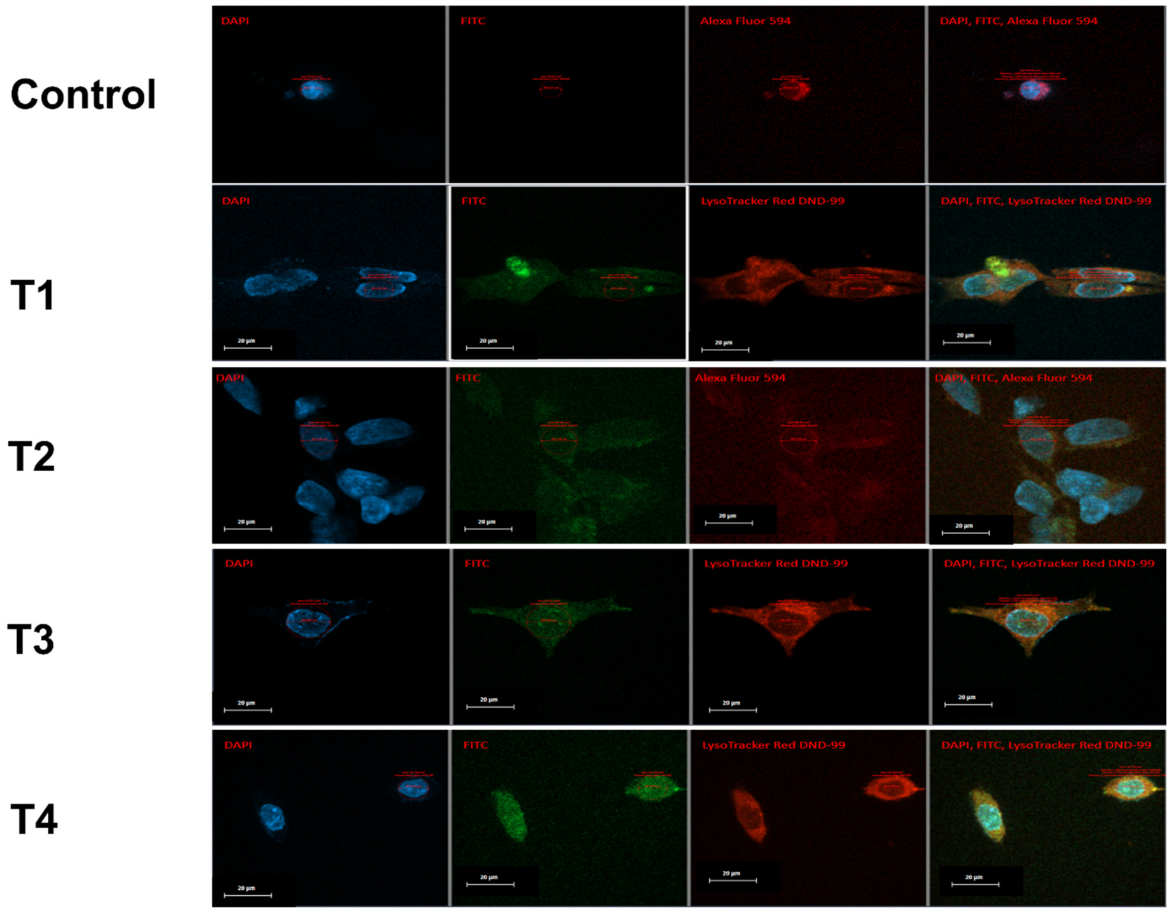

Figure S3. Uptake and localization of TiO_2 in RAW 294.7,(A) and HEK-293 (B). Confocal microscopy of cellular uptake of RAW 294.7 cells incubated for 1h at 6.26 µg/mL. Cell nuclei were stained with DAPI (blue), lysosomes were stained using LysoTracker (red) and green represent FITC-conjugated TiO_2 NPs. The shown images are representative of three independent experiments.

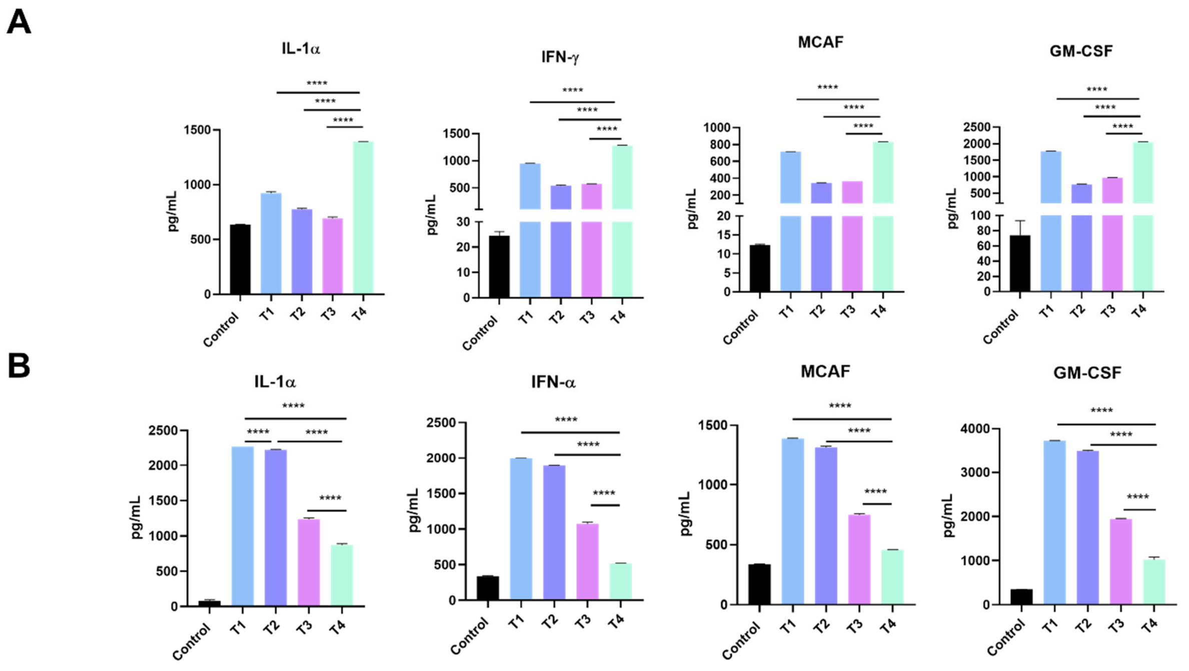

Figure S4. Inflammatory cytokines levels in (A) RAW 264.7 and (B) HEK-293 cells after exposure to TiO2 NPs in comparison to control. T1 and T4 show the highest potential to increase IL-1α, IFN-γ, MCAF, and GM-CSF levels in RAW 264.7. T1 and T2 induce the highest increase in IL-1α, IFN-γ, MCAF, and GM-CSF in HEK-293 cells. Data are represented as mean ± SD (n = 3). Statistical significance was obtained with p-values ≤ 0.05, where **** $p < 0.0001$.

Table S1 Multiple comparison test of the oxidative potential of the TiO_2 NPs and generation of ROS in RAW 264.7 (top) and HEK-293 (bottom)

	**1 h**	**2 h**	**4 h**	**6 h**	**8 h**	**12 h**	**24 hr**
**T1 vs. T2**	ns	ns	ns	ns	*	ns	*
**T1 vs. T3**	ns	ns	ns	ns	ns	ns	ns
**T1 vs. T4**	ns	ns	ns	ns	ns	ns	*
**T2 vs. T3**	ns	ns	ns	ns	ns	ns	ns
**T2 vs. T4**	ns	ns	ns	ns	*	ns	ns
**T3 vs. T4**	ns	ns	ns	ns	ns	ns	ns

	**1 h**	**2 h**	**4 h**	**6 h**	**8 h**	**12 h**	**24 hr**
**T1 vs. T2**	ns	ns	ns	ns	ns	ns	ns
**T1 vs. T3**	ns	ns	ns	ns	ns	ns	ns
**T1 vs. T4**	ns	ns	ns	ns	ns	ns	ns
**T2 vs. T3**	ns	ns	ns	ns	ns	ns	ns
**T2 vs. T4**	ns	ns	ns	ns	ns	ns	ns
**T3 vs. T4**	ns	ns	ns	ns	ns	ns	ns

Statistical significance was obtained with p-values ≤ 0.05, where * $p \leq 0.05$
